# Modulation of GLO1 Expression Affects Malignant Properties of Cells

**DOI:** 10.3390/ijms17122133

**Published:** 2016-12-18

**Authors:** Antje Hutschenreuther, Marina Bigl, Nasr Y. A. Hemdan, Tewodros Debebe, Frank Gaunitz, Gerd Birkenmeier

**Affiliations:** 1 Institute of Biochemistry, Medical Faculty, University of Leipzig, Johannisallee 30, Leipzig 04103, Germany; antje.hutschenreuther@gmail.com (A.H.); marina.bigl@medizin.uni-leipzig.de (M.B.); nhemdan@yahoo.de (N.Y.A.H.); 2 Max Planck Institute of Evolutionary Anthropology, Deutscher Platz 6, Leipzig 04103, Germany; 3 Institute of Medical Microbiology, Faculty of Medicine, University of Leipzig, Liebigstrasse 21, Leipzig 04103, Germany; TewodrosDebebe.aklilu@medizin.uni-leipzig.de; 4 College of Medicine and Health Sciences, Bahir Dar University, Bahir Dar P.O. Box 79, Ethiopia; 5 Department of Neurosurgery, University Hospital Leipzig, Liebigstrasse 20, Leipzig 04103, Germany; frank.gaunitz@medizin.uni-leipzig.de

**Keywords:** glyoxalase 1, MCF-7 cells, HEK 293 cell, aerobic glycolysis, malignant transformation, methylglyoxal

## Abstract

The energy metabolism of most tumor cells relies on aerobic glycolysis (Warburg effect) characterized by an increased glycolytic flux that is accompanied by the increased formation of the cytotoxic metabolite methylglyoxal (MGO). Consequently, the rate of detoxification of this reactive glycolytic byproduct needs to be increased in order to prevent deleterious effects to the cells. This is brought about by an increased expression of glyoxalase 1 (GLO1) that is the rate-limiting enzyme of the MGO-detoxifying glyoxalase system. Here, we overexpressed GLO1 in HEK 293 cells and silenced it in MCF-7 cells using shRNA. Tumor-related properties of wild type and transformed cells were compared and key glycolytic enzyme activities assessed. Furthermore, the cells were subjected to hypoxic conditions to analyze the impact on cell proliferation and enzyme activities. Our results demonstrate that knockdown of GLO1 in the cancer cells significantly reduced tumor-associated properties such as migration and proliferation, whereas no functional alterations where found by overexpression of GLO1 in HEK 293 cells. In contrast, hypoxia caused inhibition of cell growth of all cells except of those overexpressing GLO1. Altogether, we conclude that GLO1 on one hand is crucial to maintaining tumor characteristics of malignant cells, and, on the other hand, supports malignant transformation of cells in a hypoxic environment when overexpressed.

## 1. Introduction

Glyoxalase 1 (GLO1; *S*-d-lactoylglutathione lyase, EC 4.4.1.5) is part of the glyoxalase system, which, in addition to GLO1, consists of GLO2 (hydroxyacyl glutathione hydrolase, EC 3.1.2.6) and of catalytic amounts of reduced glutathione (GSH). It is particularly expressed at early stages of embryogenesis [1], and it is responsible for the conversion of reactive oxo-aldehydes such as methylglyoxal (MGO) to d-lactate. MGO mainly originates from the spontaneous degradation of triose phosphates during glycolysis. Under normal physiological conditions, 0.1%–0.4% of the glucose consumed by the cells and shuttled through glycolysis is transformed to MGO [2]. Because MGO is cytotoxic and can damage proteins and DNA [2,3], its detoxification is crucial for cells. Under conditions of high glycolytic flux, such as aerobic glycolysis, the formation of MGO is increased [4]. Aerobic glycolysis, i.e., the conversion of glucose to lactate in the presence of oxygen, was first observed in cancer cells by Otto Warburg [5]. His observation describes a common feature of proliferating cells [6] such as embryonic and many tumor cells [7,8,9]. Consequently, elevated levels of GLO1 were found in many human tumor tissues such as colon [10], breast [11], prostate [12], and melanoma [13] and in corresponding tumor cell lines [14], which may reflect a cellular response to increased intracellular MGO stress. High expression of GLO1, therefore, seems to be a key requirement during malignant transformation of cells. This hypothesis is supported by an enlarged gene copy number in 8.4% of human cancer tissues [15]. In addition, a positive correlation between the amount of GLO1 protein and tumor grade has been described in breast tumor biopsies [16].

Furthermore, it was shown that overexpression of GLO1 in NIH3T3 cells resulted in increased resistance to the chemotherapeutic drugs mitomycin and doxorubicin [17], while inhibiting GLO1 activity sensitized cells to chemotherapeutics [18]. It was also shown that knockdown of GLO1 led to apoptosis, accumulation of MGO and cytotoxicity [15]. A high glycolytic rate of tumors is thought to be an adaptation to intermittent hypoxia in pre-malignant lesions and it is particularly apparent in invasive metastatic tumors. Systems modeling and empirical observations indicate that enhanced glycolysis promotes unconstrained tumor proliferation, supplying precursor molecules for biomass production [6,19]. Moreover, recent data indicate that GLO1 may be a valid molecular target for cancer chemotherapy, and pharmacological inhibitors of GLO1 were shown to have anticancer effects in vitro and in vivo [20,21,22].

In the present study, we knocked-down GLO1 in the tumorigenic epithelial human breast cancer cell line MCF-7 and overexpressed it in epithelial human embryonic kidney HEK 293 cells. Enzyme activities, cell proliferation and migration, doubling time and resistance to hypoxic conditions were assessed and discussed.

## 2. Results

### 2.1. Modulation of GLO1 Enzyme Activity Impacts Downstream Glycolytic Enzymes

**Figure 1:**
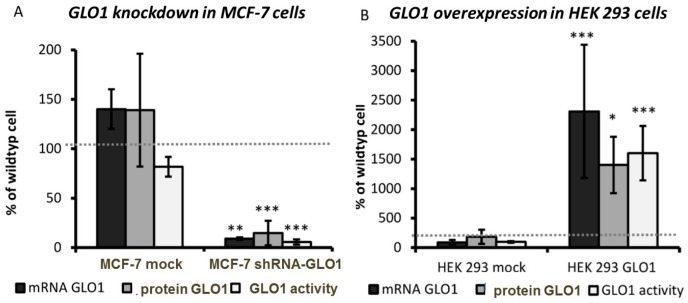

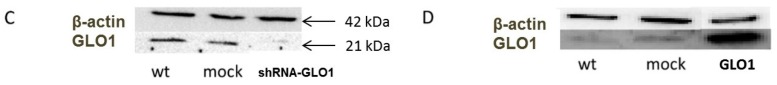
Glyoxalase 1 (GLO1)-knockdown in MCF-7 cells and GLO1-overexpression in HEK 293 cells. GLO1-mRNA expression, protein amount and enzyme activity were normalized to wild type cells (wt) and compared to mock-transfected (mock) cells. The **gray** dashed line indicates 100% of wild type cells (*n* = 3). (**A**) MCF-7; (**B**) HEK 293—the amount of GLO1 protein in cytosolic cell extracts was semi-quantitatively determined by Western blotting and normalized to β-actin as reference; (**C**) MCF-7 wild type, MCF-7 mock, MCF-7 shRNA-GLO1; protein load 40 µg; (**D**) HEK 293 wild type, HEK 293 mock, HEK 293-GLO1; protein load 20 µg). Statistical significance was determined by Student’s *t*-test with: * *p* < 0.05; ** *p* < 0.01; *** *p* < 0.001. When we analyzed the amount of ATP in cell lysates and NADPH in living cells as indicators of viability and energy metabolism, we found no significant difference between wild type and transformed cells (data not shown).

In order to evaluate the impact of GLO1 activity on the regulation of glycolytic enzymes, we established cell lines, in which GLO1 was either knocked-down (MCF-7 shRNA-GLO1) or overexpressed (HEK 293-GLO1). As shown in Figure 1A,C, GLO1-mRNA, protein and enzyme activity were significantly reduced in MCF-7 knockdown cells compared to mock-transfected cells. As expected, GLO1-mRNA, protein and enzyme activity increased more than twenty-fold in HEK 293-GLO1 cells (Figure 1B,D).

In order to assess a possible influence of GLO1 on glycolysis, we determined the activities of the three key glycolytic enzymes hexokinase (HK), phosphofructokinase (PFK) and pyruvate kinase (PK) [23]. In addition, we analyzed the enzyme activity of glucose-6-phosphate dehydrogenase (G6PDH), which is known to connect cell growth and NADPH supply via the pentose phosphate pathway [24]. Accordingly, the enzyme activities expressed as nanokatal (nkat) per mg protein are shown in Table 1. Wild type cells displayed the highest activity of PK and the lowest activity of HK. The activities of PFK and G6PDH were significantly lower in HEK 293 cells compared to MCF-7 cells.

At this point, it may be important to note that we also looked for cellular compensation mechanisms in MCF-7 shRNA-GLO1 cells. Therefore, we analyzed the activity of the NADPH-dependent α-oxo-aldehyde dehydrogenase aldose reductase [25]. However, we failed to illustrate differences in enzyme activity between wild type, mock-transfected and MCF-7 shRNA-GLO1 cells (data not shown).

### 2.2. Tumor-Related Physiological Parameters Are Affected by GLO1-Knockdown But Not by GLO1-Overexpression

**Figure 3:**
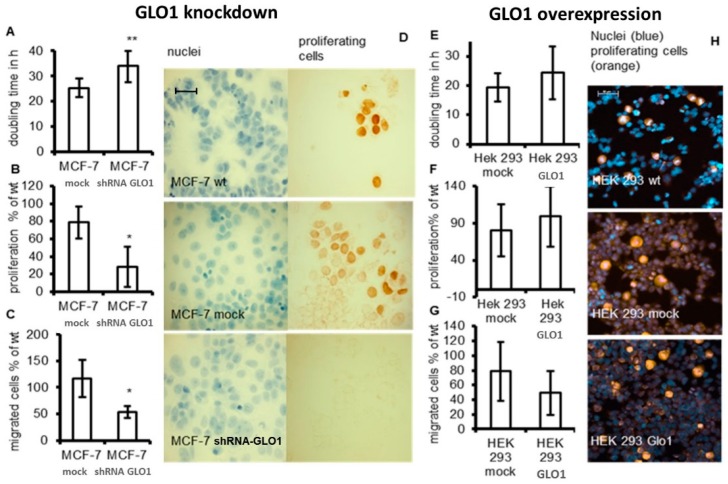
Comparison of tumor-related parameters of GLO1-knockdown and GLO1-overexpressing cells. Cell growth was analyzed by the determination of doubling time, by a proliferation assay using WST-1 reagent and by immunohistochemical staining for Ki-67. (**A**,**E**): doubling time of cells in hours; (**B**,**F**): proliferation after 24 h determined by WST-1 assay and expressed as percentage of wild type (wt); (**D**,**H**): immunohistochemical staining of the proliferation marker Ki-67; (**C**,**G**): cell migration of GLO1-knockdown and GLO1-overexpressing cells compared to wild type as determined by Boyden chamber assays. Scale bars: 50 µm. Statistical significance was determined by Student’s *t*-test with: * *p* < 0.05; ** *p* < 0.01.

To assess the impact of GLO1 expression on different tumor cell parameters, we compared the doubling time of cells, cell migration and proliferation to wild type and mock-transfected cells. Whereas the doubling time of MCF-7 shRNA-GLO1 cells was significantly increased (*p* < 0.05) from 23 (control) to 33 h (Figure 3A), no significant changes in doubling time of GLO1-overexpressing HEK 293 cells compared to the control were detected (Figure 3E). The observed unchanged doubling time in cells overexpressing GLO1 is in accordance with results of others who assessed the proliferation of NIH3T3 in a similar way [17]. As shown in Figure 3F, GLO1-overexpression in HEK 293 cells did not affect proliferation. On the contrary, GLO1-knockdown in MCF-7 cells exhibited a significantly diminished rate of proliferation (50% of control values) (Figure 3B). In addition, a lower cell number was ascertained in GLO1-knockdown cells indicated by immunostaining for Ki-67 (Figure 3D,H). Downregulation of GLO1 also abated the migration of MCF-7 shRNA-GLO1 cells to approximately 50% compared to wild-type cells, whereas overexpression of GLO1 displayed no effect (Figure 3C,G). It may be interesting to note that the potential to migrate was approximately two-fold higher in MCF-7 breast cancer cells compared to HEK 293 cells. The ability of MCF-7 tumor cells to form colonies in soft agar was used as an additional parameter of cell malignancy. Accordingly, we found that the anchorage-independent growth of MCF-7 shRNA-GLO1 cells was reduced to 50% of that of wild type cells (data not shown).

**Figure 4:**
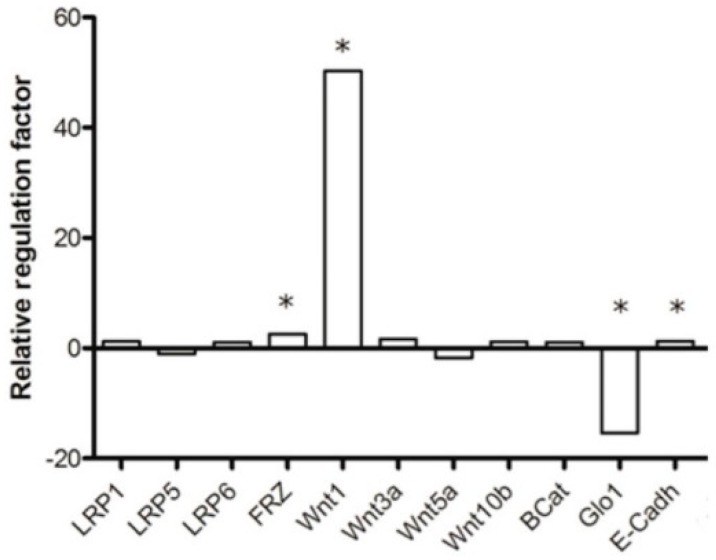
Expression of key genes of the WNT/β-catenin signaling pathway in MCF-7 shRNA-GLO1 cells. The plot shows the up- or downregulation of mRNA expression relative to mock-transfected control cells as fold-change calculated by the ΔΔ*C*t method (REST^©^2009 Software downloaded from http://www.wzw.tum.de/gene-quantification/) using the *C*t values from the genes of interest normalized to the *C*t values of GAPDH mRNA (housekeeping gene). Statistical significance was determined by Student’s *t*-test with: *n* = 3 and * *p* < 0.05.

Cell proliferation, migration and invasion are closely related to WNT/β-catenin signaling [26]. Therefore, we determined the mRNA expression of several components of the WNT/β-catenin signaling pathway, namely LRP1, LRP5, LRP6, FRZ, WNT1, WNT3a, WNT5a, WNT10b, β-catenin and E-cadherin in MCF-7 shRNA-GLO1 cells and compared it to mock-transfected control cells. We observed an approximately fifty-fold enhanced expression of mRNA encoding Wnt1 in cells with silenced GLO1 (Figure 4). A small but significant enhancement of expression was observed with regard to mRNA encoding frizzled (FRZ) and E-cadherin, whereas mRNA encoding of all other WNT/β-catenin components remained unchanged.

### 2.3. GLO1-Overexpressing Cells Show Better Adaption to Hypoxia

**Figure 5:**
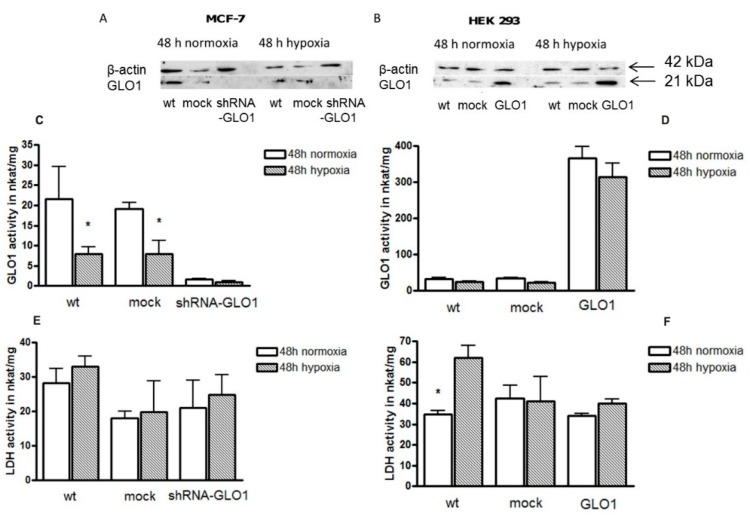
The impact of hypoxia on cells expressing different amounts of GLO1. (**A**,**B**): Western blots of cell extracts immunoblotted for GLO1 and β-actin after 48 h of culture under normoxic and hypoxic conditions. (**A**): MCF-7 control and MCF-7 shRNA-GLO1; (**B**): HEK control and HEK 293-GLO1. Each lane contained 15 µg protein; all antibodies were used at a 1:4000 dilution; (**C**,**D**): GLO1 enzyme activity; (**E**,**F**): Lactate dehydrogenase (LDH) activity under normoxic and hypoxic conditions after 48 h of incubation (*n* = 4). Statistical significance was determined by Student’s *t*-test with: * *p* < 0.05.

A high glycolytic rate of tumor cells is considered to reflect adaptation to a hypoxic environment of tumor cells [19]. Therefore, we asked whether hypoxia also influences GLO1 activity in order to compensate the deleterious effects of enhanced MGO production. Thus, GLO1 activity was measured in MCF-7 shRNA-GLO1 and HEK 293-GLO1 cells and compared to the corresponding mock-transfected control cells under normoxic and hypoxic (2% O_2_) conditions. After 24 h of incubation, no significant change of GLO1 activity was detected in any cell line under normoxic conditions (data not shown). Exposing the cells to hypoxic conditions for 48 h, GLO1 activity of MCF-7 wild type and mock-transfected control cells was significantly reduced to less than 50% of the activity of cells cultured under normal oxygen pressure (Figure 5C). Compared to MCF-7 cells, the effect of hypoxia was negligible in HEK 293 cells (in line with previous results [27]) with the exception of mock-transfected cells showing a significant reduction of GLO1 activity (Figure 5D).

It is known that lactate dehydrogenase (LDH) is upregulated in many tumor cells and is supposed to play a key role in maintaining tumorigenic properties under hypoxic conditions [28]. For this, we also measured LDH activity under normal and hypoxic conditions. In contrast to GLO1 activity, the activity of LDH after 48 h of hypoxia only slightly changed (Figure 5E) but was significantly enhanced in HEK 293 wild-type and GLO1-overexpressing cells (Figure 5F). It should also be noted that both GLO1 and LDH activity were higher in HEK 293 cells than in MCF-7 cells.

**Figure 6:**
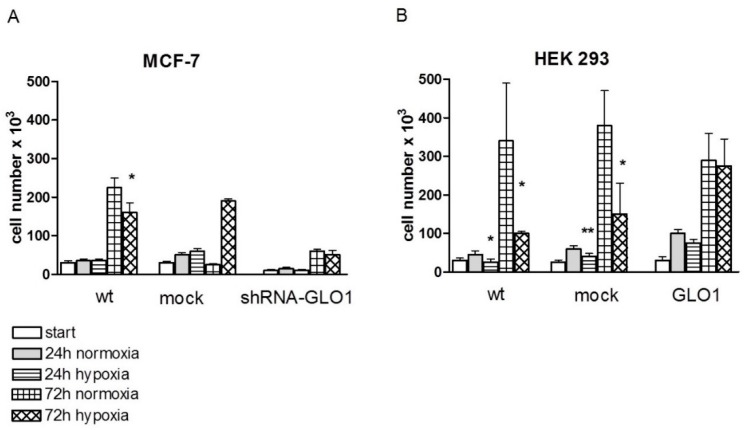
Cell growth after 24 h and 72 h under hypoxic and normoxic conditions. (**A**): MCF-7 cells; (**B**): HEK 293 cells (*n* = 4). Statistical significance was determined by Student’s *t*-test with: * *p* < 0.05; ** *p* < 0.01.

In addition to the determination of enzyme activities, we also compared cell growth under normoxic and hypoxic conditions after 24 and 72 h of incubation. We found that the growth of HEK 293 wild-type and HEK 293 mock-transfected cells were considerably inhibited by hypoxia, whereas wild-type and mock-transfected MCF-7 cells were less affected (Figure 6).

Hypoxia had no effect on cell growth of MCF-7 cells after 24 h of incubation (Figure 6A), whereas cell growth was significantly reduced to 50%–70% in HEK 293 wild-type and HEK 293 mock-transfected cells within this time interval (Figure 6B). After 72 h of culture, the cell number under hypoxic conditions was reduced in all cells (wild-type and mock-transfected cells) except for the cell lines containing modulated GLO1 activity (MCF-7 shRNA-GLO1 and HEK 293-GLO1) (Figure 6A,B). While cell growth of wild type and mock-transfected MCF-7 cells was reduced by ~20% under hypoxic conditions, the growth of HEK 293 wild type and mock-transfected cell was reduced to less than 50% compared to growth under normoxia. HEK 293 cells overexpressing GLO1 exhibited no inhibition of cell growth under hypoxic conditions (Figure 6B). Interestingly, these cells, even under normoxic conditions, had the highest proliferation rate compared to all other cells, which may indicate that overexpression of GLO1 may have a general positive effect on cell viability.

## 3. Discussion

Here, we analyzed the influence of knockdown and overexpression of the MGO-degrading enzyme glyoxalase 1 (GLO1) on tumor-associated parameters in the epithelial breast cancer cell line MCF-7 and in the human embryonic kidney cell line HEK 293. Although HEK 293 is not a bona fide non-cancerous cell-line, it is a good model to analyze the effects of overexpression of GLO1 on different physiological parameters, as it was possible to enhance the enzymatic activity of GLO1 by stable transfection more than twenty-fold compared to mock-transfected cells. It is widely accepted that glycolytic enzymes and metabolites including MGO are involved in the regulation of tumor cell metabolism, proliferation and survival [29,30]. Overall, we have shown that GLO1-knockdown modulates the activity of glycolytic enzymes such as PK and tumor-associated properties of cells such as migration and proliferation, while overexpression of GLO1 did not affect these parameters, but rather resulted in a better adaption of cells to hypoxia.

We found that PK was the only enzyme whose activity was significantly reduced as a consequence of GLO1-knockdown in MCF-7 tumor cells. PK is a key regulating enzyme of the glycolytic flux that is known to have four isoforms in humans: L, R, M1 and M2. Whereas the L and R isoforms are exclusively expressed in liver and red blood cells [31,32], M1 is mostly expressed in adult tissue, and M2 is expressed solely in rapidly proliferating tissues such as tumor tissue [23,33]. The latter is highly expressed in MCF-7 breast cancer cells [31]. It was shown that inhibition of PKM2 caused downregulation of the glycolytic flux in cancer cells and resulted in growth inhibition [31,34,35].

We demonstrated that knockdown of GLO1 results in a significant reduction of cell proliferation and cell migration. Growth arrest of tumor cells resulting from GLO1 inhibition was shown to be associated with elevated levels of MGO [17,21]. Silencing of GLO1 in tumors with high rates of glycolysis has been shown to cause a marked accumulation of MGO and cytotoxicity [15]. Anti-proliferative effects of MGO were also found after exogenous supply of the compound [22,29]. GLO1-knockdown was found to increase expression of the receptor for advanced glycation end products (RAGE) and to enhance the intracellular modification of proteins by MGO [36]. According to our results, an alternative degradation of MGO in GLO1-knockdown cells seems unlikely as the activity of aldose reductase; the second important MGO-detoxifying enzyme was not elevated. Although GLO1-knockdown was accompanied by reduced cell proliferation, we could not observe the reverse, namely increased cell growth as a result of GLO1-overexpression in HEK 293 cells.

As shown by others, a reduced expression of GLO1 using shRNA led to a significant inhibition of cell growth and induction of apoptosis in primary cultured cells from hepatocellular carcinoma (HCC) with an amplified GLO1 gene, whereas no inhibitory effect on cell proliferation was observed in HCC cells with normal GLO1 gene copy numbers [37,38]. This indicates that the responsiveness to GLO1 silencing strongly depends on the expression level of this enzyme in cells. This is also in agreement with our previous observation, that ethyl pyruvate, a competitive inhibitor of GLO1, strongly inhibited the growth of monocytic leukemic THP-1 cells, while being harmless to parental normal human blood monocytes exhibiting seven-fold lower expression of GLO1 than THP-1 cells [39]. This is in line with earlier experiments showing that metabolically activated white blood cells are more prone to GLO1 inhibition than non-activated cells [40].

A common feature of tumor cells is their high glycolytic flux. In line with our findings, it seems conclusive that inhibition of GLO1 may be a therapeutic option for patients with tumors exhibiting a high level of GLO1 activity. In fact, cancer cell lines with an amplification of the *GLO1* gene were shown to have a higher sensitivity to the growth inhibitory effect of a potent GLO1 inhibitor [15].

Detecting a correlation between GLO1-knockdown and cell migration, we assessed mRNA expression levels of WNT-1/β-catenin pathway components known to be involved in cell proliferation, migration, and differentiation [41]. Surprisingly, we found a very strong increase in expression of WNT-1 in MCF-7 shRNA-GLO1 cells, and a lower but significant elevation of FRZ and E-cadherin mRNA. Upregulation of WNT-1 was also found after cardiac injury due to ischemia [42]. Ischemia is known to enhance MGO levels in cells and to decrease the activity of GLO1, which could lead to reduced migration [27]. On the contrary, inhibition of the WNT/β-catenin pathway was found to decrease cell growth, migration and invasion [43]. These contradictory effects could indicate cell type specific differences.

Overexpression of GLO1 is supposed to be a crucial step towards the malignant transformation of cells accompanying changes of energy metabolism and the development of drug resistance [4]. However, overexpression of GLO1 does not a priori lead to enhanced cell growth because it did not affect migration and proliferation as shown in Figure 3.

This situation changed upon culturing cells under hypoxic conditions. When different leukemic cell lines were subjected to hypoxia, cell cycle arrest in the G1 phase was observed already after two days, which was followed by apoptosis within seven days. The few cells that survived long-term culture under hypoxia exhibited increased amounts of GLO1 protein and enhanced enzymatic activity [44]. In our experiments, we confirmed these results in another cell model by demonstrating that overexpression of GLO1 in HEK 293 cells prevented hypoxia-induced growth inhibition. GLO1-overexpression diminished levels of advanced glycation end-products (AGEs) and reduced oxidative stress in diabetic rats [45]. It also attenuated carbonyl stress and retarded senescence in renal cells in vitro and in vivo [46]. Furthermore, it was found that overexpression of GLO1 in rats prevented histological and functional damage after renal ischemia-reperfusion injury [27]. This protective effect of GLO1-overexpression was associated with a decreased level of MGO, reduction of oxidative stress and reduced tubular cell apoptosis. Taken together, these results show, that overexpression of GLO1 may be crucial to help cells surviving under hypoxic conditions.

Unexpectedly, GLO1 activity was inhibited under hypoxic conditions in all three MCF-7 cell types (wild-type, mock, shRNA-GLO1). GLO1 expression is under the control of Nrf2 [47] and its activity is metabolically regulated through reversible glutathionylation [48]. Oxidative stress, as caused by hypoxia, could decrease the cellular availability of glutathione, resulting in reduced GLO1 activity, elevated MGO and increased amounts of reactive oxygen species. At this point, it is also interesting to note that resistance to therapeutic drugs was shown to be associated with an increased sensitivity towards toxic dicarbonyls and reduced amounts of free sulfhydryl groups, both affected by the activity of GLO1 [49]. Survival advantages of malignant cells compared to normal cells under hypoxic conditions as described in [50,51] displayed elevated LDH activity under hypoxia similar to our results. This had to be expected as LDH expression is regulated by the hypoxia-inducible factor (HIF-1) [52,53].

In conclusion, we demonstrate that GLO1-knockdown in MCF-7 cells resulted in a reduction of cell proliferation and migration, while overexpression of GLO1 in HEK 293 cells revealed a better adaption to hypoxic growth conditions, which are crucial during tumor formation.

## 4. Materials and Methods

### 4.1. Chemicals

Glass beads (0.25–0.50 mm), GSH, protein marker Roti^®^-Mark, Rotiphorese Gel 30 SDS Ultra-Pure and Trishydroxyaminoethane (Tris) were from Roth (Karlsruhe, Germany), Cell Titer-Glo^®^ reagent and Cell Titer-Blue reagent^®^ were from Promega, (Mannheim, Germany), Coommassie Brilliant Blue R250, *N*,*N*,*N′*,*N′*-tetramethylethylenediamine (TEMED), 3.3-diaminobenzidin-4-hydrochloride (DAB) and β-mercaptoethanol were from Serva (Heidelberg, Germany) and WST-1 reagent was from Roche (Mannheim, Germany). Transfection reagent TurboFect™ was from Fermentas (St. Leon-Rot, Germany), RPMI 1640, Dulbecco’s modified Eagle Medium (DMEM), Opti-MEM^®^, fetal calf serum (FCS) and Zeocin were from Invitrogen (Karlsruhe, Germany), hematoxylin was from Merck (Darmstadt, Germany), milk powder from Heirler Cenovis (Radolfzell, Germany), and bovine serum albumin was from PAA Laboratories (Linz, Austria). Cell lines used were the human breast adenocarcinoma cell line MCF-7 (ATCC HTB-22) and the human embryonic kidney cell line HEK 293 (ATCC CRL-1573). All additional cell culture material was from Greiner Bio-One (Frickenhausen, Germany). All other chemicals were from Sigma-Aldrich (Taufkirchen, Germany).

### 4.2. Cell Culture

Cells were cultured in RPMI 1640 (2 g/L) (MCF-7) or DMEM (4.5 g/L glucose) (HEK 293) medium supplemented with 10% fetal calf serum (FCS) and penicillin/streptomycin (100 U penicillin/mL; 100 mg streptomycin/mL) at 37 °C under 5% CO_2_ in humidified atmosphere using an incubator (Hera Cell 150 Heraeus, Hanau, Germany). Medium of cells transfected with the plasmid pTER-EGFP containing either scrambled DNA for mock-transfection (MCF-7 mock) or GLO1 specific siRNA (MCF-7 shRNA-GLO1) or pcDNA3 without insert (HEK 293 mock) or a sequence encoding GLO1 (HEK 293-GLO1) contained additionally 250 µg/mL Zeocin. Cells were grown in 25 cm^2^ cell culture flasks and sub-cultured using trypsin/EDTA solution. Cell vitality was determined by the trypan blue exclusion assay. Cell culture under hypoxic conditions was performed in 6-well plates (enzyme activity) and 24-well plates (determination of doubling time), respectively. Cells were synchronized by keeping cells in the absence of FCS for 24 h. For hypoxia studies, cells were contained in a hypoxia chamber with 2% O_2_.

### 4.3. Protein Extraction and Determination of Protein Content

Cytosolic protein extracts used for the analysis by Western blots and enzyme activity measurement of GLO1, glucose-6-phosphat dehydrogenase (G6PDH) and lactate dehydrogenase (LDH) were prepared using extraction buffer composed of 25 mM Tris/HCl, 2 mM EDTA, 2 mM DTT, 1 mM PMSF, 10% glycerol, 1% Triton X100, pH 8.0 supplemented with 0.3% protease inhibitor cocktail. Protein content of samples was determined in triplicates according to Bradford [54]. Cell lysates for the determination of enzyme activity of phosphofructokinase (PFK), hexokinase (HK) and pyruvate kinase (PK) were prepared using glass beads in homogenization buffer containing 50 mM Tris/HCl pH 8.5, 50 mM NaF, 1 mM EDTA, 1 mM ATP, 1 mM Fructose 6-phosphate, 10 mM dithiothreitol, 1 mM phenylmethylsulfonyl fluoride and 1 µM E64. Samples were vortex mixed five times 20 s each, and supernatant was centrifuged at 4 °C, 12,000× *g* for 40 min.

### 4.4. Construction of shRNA Expressing Plasmids for GLO1 Silencing

Human GLO1 specific shRNA was designed as 63-mer containing a hairpin-loop and cloned into pSuper vector with the H1 RNA polymerase promoter. The original vector using an inducible system for stable integrated siRNA and an EGFP cassette was obtained as in [55]. A Zeocin-resistance cassette allowed stably transfected eukaryotic cells to be selected. The oligonucleotides encoding the Glo1-siRNA were *shGlo1 For*: 5′-GATCCCGCATCTAGGACTGATGGATTTCAAGAGAATCCATCAGTCCTAGATGCTTTTTGGAAA-3′ and *shGLO1 Rev* 5′-AGCTTTTCCAAAAAGCATCTAGGACTGATGGATTCTCTTGAAATCCATCAGTCCTAGATGCGG-3′. As control a scrambled RNA was used and also cloned into the vector. *Negfo*: 5′-GATCCCAGTACTGCTTACGATACGGTTCAAGAGACCGTATCGTAAGCAGTACTTTTTTGGAAA-3′; *Negre*: 5′-AGCTTTTCCAAAAAAGTACTGCTTACGATACGGTCTCTTGAACCGTATCGTAAGCAGTACTGG-3′. Negative control siRNAs were obtained from ThermoFisher Scientific (Rockford, IL, USA) and then transferred to shRNA. Negative shRNAs have no significant sequence similarity to mouse, rat, or human gene sequences as analyzed by BLAST search. The oligonucleotides were annealed and subcloned downstream of the H1 promoter in pTER-EGFP using *Hin*dIII and *Bgl*II. All sequences were subjected to NCBI Blast query to confirm the lack of homology to other known genes. Plasmids were sequenced using the BigDye terminator cycle sequencing kit (Qiagen, Hilden, Germany) and the ABI Prism genetic analyzer Model 377 (Applied Biosystems, Darmstadt, Germany).

### 4.5. Cloning of GLO1-Overexpressing Plasmids

Full length human GLO1 cDNA was generated by RT-PCR and sub-cloned into pCR 2.1 TOPO vector from the TOPO TA Cloning Kit (Invitrogen, Waltham, MA, USA) according to the manufacturer’s instructions. The transcript was digested with *Eco*RI and *Xb*aI and inserted into pcDNA3.1/Zeocin plasmid vector (Invitrogen, Waltham, MA, USA), respectively. Both constructs were confirmed by sequencing and digest.

### 4.6. Generation of Stably Transfected Cell Lines

Transfection was conducted using TurboFect™ according to the manufacturer’s instructions. Briefly, cells were seeded in 6-well plates and cultured over night until 50%–70% confluence. Cultivation medium was replaced by Opti-MEM and cells were transfected immediately with a mixture of 4 µg DNA in 1 mL Opti-MEM and 6 µL TurboFect™ incubated for 20 min at RT. Cells were incubated 5 h before Opti-MEM medium was replaced by normal cultivation medium. After two days, cells were detached by trypsin, split to ~20% confluence, and transfected cells were selected with Zeocin and by EGFP fluorescence.

### 4.7. Total RNA Isolation and RT-PCR

Total RNA was isolated using RNeasy^®^ (Qiagen) according to the manufacturer’s instructions and purified with RNase-Free DNase to eliminate genomic DNA. For probe-based RT-PCR, LightCycler^®^ 480 Probes Master (Roche) and the following sense (s) and antisense (as) primer sequences were used: (i) *GAPDH* (NM_002046.3, Roche Universal Probe Library—UPL # 60): s: 5′-AGCCACATCGCTCAGACAC-3′, as: 5′-GCCCAATACGACCAAATCC-3′; (ii) *β-actin* (NM_001101.3, UPL # 64): s: 5′ CCAACCGCGAGAAGATGA 3′, as: 5′ CCAGAGGCGTACAGGGATAG 3′; (iii) *Eukaryotic translation elongation factor 2* (*EEF2*. NM_001961.3, UPL # 25) s: 5′-CTG GAGATCTGCCTGAAGGA-3′, as: 5′-GAGACGACCGGGTCAGATT-3′; (iv) *GLO1* (NM_006708.2, UPL # 84): s 5′-CCCCAGTACCAAGGATTTTCT-3′, as 5′-TGGGAAAATCACATTTTTGGA-3′; (v) *LRP1* (NM_002332.2, UPL # 83) s: 5′-GATGAGACACACGCCAACTG-3′, as: 5′-CGGCACTGGAACTCATCA-3′; (vi) *LRP5* (NM_002335.2, UPL # 23) s: 5′-GAACATCAAGCGAGCCAAG-3′, as: 5′-TGGCTCAGAGAGGTCAAAACA-3′; (vii) *LRP6* (NM_002336.2, UPL # 71) s: 5′-ATCCGAAAGGCACAAGAAGA-3′, as: 5′-GACTCGGAACTGAGCTCA CAA-3′; (viii) *Frizzled-related protein* (*FRZ*, NM_001463.2, UPL # 31) s: 5′-TCATGGGCTATGAAGATGAGG-3′, as: 5′-TCATATCCCAGCGCTTAACTT T-3′; (ix) *WNT1* (NM_005430.2, UPL # 81) s: 5′-CGCTGGAACTGTCCCACT-3′, as: 5′-AACGCCGTTTCTCGACAG-3′; (x) *WNT3A* (NM_033131.2, UPL # 64) s: 5′-AACTGCACCACCGTCCAC-3′, as: 5′-CCGACTCCCTGGTAGCTTT-3′; (xi) *WNT5A* (ENST00000264634.4, UPL # 11) s: 5′-TAAGCCCAGGAGTTGCTTTG-3′, as. 5′-CTGAACAGGGTTATTCATACCTAGC-3′); (xii) *WNT10B* (U81787.1, UPL # 27) s. GCGAATCCACAACAACAG G-3′, as: 5′-TCCAGCATGTCTTGAACTGG-3′; (xiii) *β-catenin* (X87838.1, UPL # 21) s: 5′-GCTTTCAGTTGAGCTGACCA-3′, as: 5′-AAGTCCAAGATCAGCAGTCTCA-3′ and (xiv) *E-cadherin* (AB025105.1, UPL # 35) s: 5′-CCCGGGACAACGTTTATT AC-3′, as: 5′-GCTGGCTCAAGTCAAAGTCC-3′. REST^©^ 2009 software (http://www.gene-quantification.info/) based on an efficiency corrected mathematical model and a pairwise fixed reallocation randomization test was used to estimate gene expression relative to controls. Reactions started by an initial activation step for 10 min at 95 °C, and each following cycle consisted of a denaturation step for 10 s at 95 °C, amplification for 27 s at 60 °C, acquisition for 3 s at 72 °C, and, finally, cooling for 10 s at 40 °C. β-actin was chosen as a reference housekeeping gene, as it showed amplification efficiency similar to those of other cytokine genes and was proved to be the most stable gene in comparison to *GAPDH* and *EEF2* applying geNorm (downloaded from www.gene-quantification.com).

### 4.8. Western Blot Analysis

Equal amounts of protein were subjected to SDS-pore gradient polyacrylamide gel electrophoresis (SDS-PAGE; 4% to 20%) and run under denaturing conditions. Proteins were blotted to cellulose nitrate membranes (Whatman Schleicher & Schuell, Dassel, Germany) and GLO1 was detected by anti-GLO1 monoclonal antibodies (1 µg/mL; 1:4000) (BioMac, Leipzig, Germany) in combination with goat anti-rabbit Ig-HRP (1:4000) (Dianova, Hamburg, Germany). For comparison, β-actin was analyzed using rabbit anti-β-actin Ig (1:4000) (Acris, Hiddenhausen, Germany) in conjunction with HRP-labeled goat anti-rabbit Ig. Band visualization was performed either by using 1 mM DAB and 0.03% H_2_O_2_ as substrate for HPR reaction or by chemiluminescence detection (ThermoFisher Scientific, Rockford, IL, USA) according to the manufacturer’s instructions.

### 4.9. Immunohistochemistry

Cells were grown on chamber slides coated with poly-l-lysine, fixed with 2% paraformaldehyde in PBS and stored at −20 °C. Slides were incubated 1 h with 5% low fat milk in TBS (50 mM Tris/HCl, 150 mM NaCl, pH 7.5) containing 0.3% Triton X100 at room temperature for immunohistochemical staining. After removal of the blocking solution, the slides were incubated with Ki-67 proliferation marker monoclonal antibody according to the manufacturer’s instructions (Abcam, Cambridge, UK). Slides were washed twice with TBS 0.1% Triton X100 before incubation with the secondary antibody for 1 h was started. Visualization was performed using either a Dako REAL™ Detection System, Peroxidase/DAB+, and Rabbit/Mouse Kit (Dako, Hamburg, Germany) according to the manufacturer’s instructions or Cy3-labeled goat anti-mouse antibody (Abs550/Em570nm) (Dianova, Hamburg, Germany). Cell nuclei were stained using DAPI (1 µg/mL in TBS) (Abs358/Em461nm) for 20 min at RT or hematoxylin for 10 min at RT. After washing twice, slides were dried and coverslips were mounted using Entellan. Stained cells were evaluated with a confocal laser scanning microscope (LSM510 META, Carl Zeiss, Jena, Germany).

### 4.10. Doubling Time, Proliferation and Vitality Assay

Cell proliferation was assessed using the WST-1 assay. Five thousand cells per well in 100 µL medium were seeded in two 96-well plates. After 24 and 48 h cultivation time, 100 µL medium and 12 µL WST-1 reagent were added to each well. Absorption at 450 and 620 nm was measured for four consecutive hours. Vitality of cells was assessed using the trypan blue exclusion method as described above, and growth rate of cells was assigned by counting cells in time intervals of 24, 48 or 72 h.

### 4.11. Determination of ATP in Cell Lysates and NADPH Production

ATP was determined by means of the CellTiter-Glo^®^ Luminescent Cell Viability Assay according to the manufacturer’s instructions. 10,000 cells/100 µL medium were dispensed into the wells of a 96-well plate, allowed to settle for 24 h, followed by addition of CellTiter-Glo reagent (100 µL) using the respective Promega protocols. Ten minutes later, the amount of ATP was determined by luminescence using a Mithras microplate reader (Berthold Technologies, Bad Wildbad, Germany). For assessing the production of NADPH, the CellTiter-blue^®^ Cell Viability Assay was employed. Therefore, CellTiter-Blue (CTB) assay reagent (16 µL) was added to 10,000 cells dispensed into the wells of a 96-well plate in 100 µL of medium. The relative amount of NADPH was recorded as fluorescence developed after incubation (90 min) in an incubator using a SpectraMax M5 microplate reader (Molecular Devices, Biberach, Germany).

### 4.12. Migration Assay

Cell movement was evaluated in Boyden chambers using 24-well culture plates equipped with 8-μm pore ThinCerts. Cells (4 × 10^4^ in 100 µL RPMI/DMEM, 0.5% FCS) were seeded in the upper compartment to adhere overnight. To start cell migration, 800 µL cell medium (10% FCS) was added to the bottom well. After 4 h incubation, cells were fixed for 45 min with Karnowski solution. Then, non-migrating cells were carefully removed using cotton swabs and migrated cells on the bottom side were stained for 1 h with 800 µL hematoxylin. ThinCerts were stored in PBS at 4 °C, and the migrated cells were counted under a light microscope (magnification: 400×).

### 4.13. Anchorage-Independent Growth

Six-well plates were coated with 1.5 mL of 0.6% basal agar (Seakem LE Agarose; Lonza, Köln, Country) and 2× RPMI-FCS (1:1). This base layer was overlaid with 2.5 × 10^5^ to 1 × 10^6^ cells/well in a mixture of 0.4% agar and 2× RPMI/20% FCS (1:1). Cultures were evolved for 14 days and supplemented every second day with 100 µL RPMI-FCS. Subsequently, the plates were stained with 0.5 mL of 0.005% crystal violet and colonies of >20 cells were counted.

### 4.14. Enzyme Activity Measurements

PFK activity was measured as in [56]. Determination of hexokinase activity was conducted as in [57]. PK activity was measured using imidazole/HCl buffer, 0.1 mM NADH, 1.2 mM PEP, 1.2 mM ADP, 0.5 mM fructose diphosphate and excess of LDH. LDH was measured in buffer composed of 50 mM phosphate pH 7.0, 0.015 mM NADH and 0.2 mM pyruvate. For G6PDH, the reaction mixture contained 0.5 mM glucose 6-phosphate and 0.11 mM NADP in 960 µL Tris/KCl buffer (100 mM Tris, 100 mM KCl, 7.5 mM MgSO_4_, pH = 7.6). GLO1 activity was determined as in [58]. Enzyme activity of aldose reductase was measured as in [59]. All enzyme activities were expressed in nanokatal (nkat)/mg protein.

### 4.15. Statistical Analysis

Unless indicated otherwise, the results are presented as means ± S.D. of at least three independent experiments. Data were analyzed with Microsoft Excel 2010^©^ using the unpaired two-sided Student’s *t*-test (* = *p* < 0.05; ** = *p* < 0.01; *** = *p* < 0.001).

## 5. Conclusions

In the present study, we show that knockdown of GLO1 in MCF-7 tumor cells significantly reduced the malignant properties of the cells, characterized by the reduction of cell proliferation, cell migration and anchorage-independent growth. Concomitant reduction of PK activity indicated a reduced glycolytic flux in response to increased MGO or shortage of glutathione. Our data indicate that overexpression of GLO1 could be one key step during malignant transformation of cells, since it is not disadvantageous under normoxic conditions, but rather turns out to be beneficial under hypoxia. Our results emphasize that GLO1 can be a promising target for new cancer treatment strategies.

## Figures and Tables

**Figure 2:**
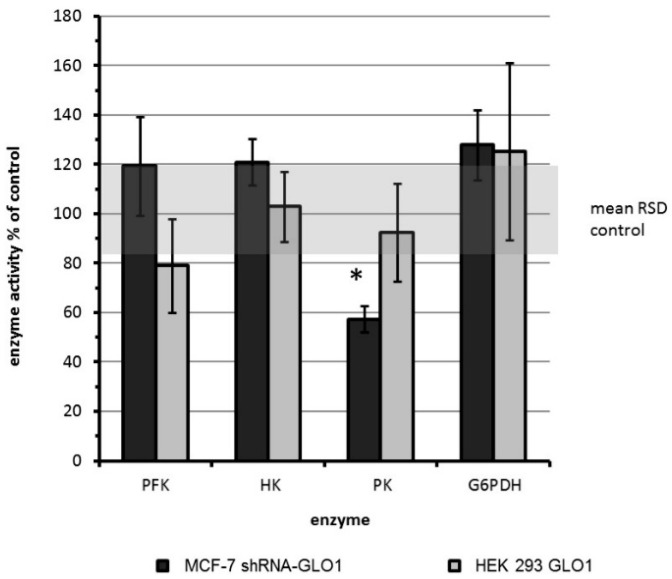
Comparison of enzyme activities of genetically modified MCF-7 and HEK 293 cells compared to the activity in mock-transfected control cells (set as 100%). The **gray** shaded area illustrates the mean relative standard deviation (RSD) of the activities in control cells. Enzyme activities of wild type and mock-transfected cells showed no significant differences (*n* = 4; data not shown).Statistical significance was determined by Student’s t-test with: * *p* < 0.05.

**Table 1:** Enzyme activities in MCF-7 and HEK 293 wild type cells expressed as nanokatal (nkat) per mg protein of cytosolic cell extracts (±SD; *n* = 4).

Activity in nkat/mg	MCF-7	HEK 293
PFK	5.83 ± 0.33	1.76 ± 0.60
HK	0.83 ± 0.16	0.98 ± 0.16
PK	95.5 ± 10.0	28.3 ± 8.3 *
G6PDH	14.8 ± 1.50	1.75 ± 0.3

The activity of the above enzymes in GLO1-knockdown and GLO1-overexpressing cells relative to their non-transfected control cells is shown in Figure 2. No significant differences between GLO1-overexpressing HEK 293 cells and HEK 293 wild type cells were observed. In contrast, a significant reduction of PK activity to approximately half of the activity of the wild type cells was found in shRNA-GLO1 cells. All other enzyme activities remained unchanged. Statistical significance was determined by Student’s *t*-test with: * *p* < 0.001.
